# Burnout

**Published:** 2019-11-28

**Authors:** Manish Ranpara

**Affiliations:** 1 University of Toronto, Ontario, Canada

I run up and down the corridor

Ticking tasks off my list

Floor to floor

Is there is anything I have missed?

Potassium - check, fluids – check

It’s my anniversary, did I message my wife?

I’m a wreck

Sleep is scant

And the hours long

This isn’t a rant

Perhaps I’m not as strong?

My stomach has an ache

I skipped dinner

I really need a break

Will things get easier for this beginner?

Surely, I am not alone

Other doctors feel like this

And it should be known

If there is light in this abyss

I do it for life

Shocking rhythms that are flat

Using medications and the surgical knife

Who else can say that?

Optimism overwhelms me

I can push through this night

Better skilled shall I be

The end is in sight

The pager rings loud

But before I go fleeing

I’ll arrange to meet my crowd

To look after my well being.

